# Use of Cathartics

**Published:** 1861

**Authors:** 


### USE OF CATHARTICS.—DR. WARE'S LECTURES.

That the alimentary canal should be well evacuated at the beginning of any considerable disease, was stated at the beginning of this lecture. A good many questions arise, however, in connection with this point, which require further remark, particularly the connection with the matters evacuated may have had with the causation of the disease. When undigested food, or food become acid, copious fæces, especially of bad appearance, bile and secretions are discharged, we are apt to infer that these have brought on the attack, and particularly if their evacuation be followed by relief. This may be so, or it may not. When a patient has recently taken food, and when, either spontaneously or by the operation of medicine, he throws it up undigested or sour, or passes it in this state from the bowels, it is a natural inference that the food has caused the attack, especially when it has been erroneous either in quality or quantity. But this inference will often be a mistaken one. It may be that the indigestion has been the consequence of disease and not its cause.

When the food has been really the sole cause of the attack, its complete evacuation is usually followed by entire relief, whether effected soontaneously or by medicine.

But the evacuation of the offending food, either by vomiting or purging, is not always complete, and portions may remain behind, keeping up a continued state of irritation in the stomach, or even the whole of the alimentary canal, indicated by nausea, retching, epigastric distress, ineffectual attempts to vomit and imperfect action of the bowels. Where this state exists it is not well to persevere in active measures to procure evacuations, but to leave the organs at rest, administering only mild, soothing and liquid nourishment, and a few grains of carbonate of soda in solution, or a small quantity of lime water, every few hours. Sometimes very soon, or at farthest after a few days, the disturbance will subside, the organs rally and relieve themselves of their contents; or if not, may be made to do so by a mild evacuant, such as rhubarb or castor oil.

Where food has not been the whole cause, its evacuation, though giving much immediate relief to the sensations of the patient, does not relieve the disease, though removing the cause of its aggravation and continuance. Both of these contingencies are well illustrated by what happens in common cholera morbus. An attack of this disease may take place simply from irritating food at any period of the year, but especially in the summer, when the organs have become irritable from the influence of heat. In such cases, promoting effectual vomiting by warm diluents, or producing it by an emetic of ipecac, is generally sufficient. If the irritation and distress is great, the addition of ten or fifteen drops of laudanum and a teaspoonful of cinnamon to the emetic will render its operation easier and more effectual, by preventing that unequal and irregular action of the stomach and bowels which prevents in an irritable organ a complete effectual effort. But there is another class of cases, occurring almost exclusively after a period of very hot weather, in which the attack is of a different kind and its cause lies deeper. In these, food does not cause the attack; the attack finds the food in the stomach and its digestion is arrested. Its presence increases the irritation of the organ, for its digestion being arrested and the condition of the stomach changed, it becomes a foreign substance. Still its evacuation does not give any relief, and violent symptoms follow, such as epigastric distress, with severe ineffectual retchings and cramps. A very early emetic, with an opiate, largely diluted, will sometimes succeed here, but more commonly the best method is to quiet all activity by very full successive doses of laudanum—from 80 to 100 drops—each dose to be repeated at once if rejected, and then leave the patient for a time without further medication. There is a series of cases lying between these two extremes. A man in health before going to bed eats largely of some unusual and improper food, and is waked up in the night by violent vomiting and purging. Another takes only his ordinary food, but is also attacked with equal or greater violence. The first throws up his food in an undigested state, and in twenty-four hours is well. The second also throws up his food in the same condition, but without the same relief. He has pain, nausea and epigastric distress, great prostration, painful spasms in the muscles of the abdomen and limbs, violent retchings with discharge of acid secretions and of green and yellow bile, perhaps copious discharges of similar matters from the bowels, he is reduced to a state of great exhaustion, becomes cold and almost pulseless—he may even die, though this is uncommon. At any rate, after the urgent symptoms have ceased, he continues ill for many days, and slowly recovers.

No doubt improper food alone will sometimes produce continued disease; but more commonly when disease is apparently produced by food, as indicated by its expulsion in an undigested or chemically altered state, there has existed a preceding predisposition which the food simply calls into activity.—There are some persons, indeed, with a stomach so weak and irritable that they may be regarded as always in such a state of predisposition. By great care they may keep themselves infinitely in a comfortable state of health, but upon the occasion of some error or diet, or from any cause of irritation or exhaustion, they will undergo an attack, not relieved by evacuation, but following a course more or less like those just described.

These remarks are intended to illustrate conditions of the stomach which may arise in disease and in any period of disease—in which some offending load is present which may be the sole cause of the symptoms from which the patient suffers, or which may be only the consequence of a condition under which the stomach has previously labored. The general principles of treatment will be always essentially the same. In either case, the removal of the load is to be desired and attempted. If the attempt is successful, in the one case the whole trouble is removed, in the other an impediment to its removal by the efforts of Nature is taken away. If unsuccessful, the organ is not to be annoyed by repeated efforts, but either to be left to rest, or its irritation soothed by palliating measures.

Much caution is to be used in judging of the state of the patient as connected with the presence of undigested food. Thus periodic attacks—such as epilepsy and sick headache—are apt to be attributed to this cause, because, either spontaneously or by an emetic, articles last eaten are rejected, and often in what is regarded as an unnatural state. Now even the contents of a stomach engaged in the act of a healthy digestion are often acid, and have a disagreeable odor and appearance; so that if a fit of epilepsy comes on during this act, and an emetic be given, it is apt to be inferred that the food has been the cause of the fit, and that it was of an improper kind. This seems to the observer to be confirmed by the fact that relief follows the act of vomiting, and as a eonsequence the articles taken are in future forbidden. Hence, a patient is sometimes unnecessarily restricted as to his diet, from the delusive idea that indigestion is the whole cause of his malady, while, in fact, the indigestion is caused by the approach of the fit. A similar remark is true of sick headache. No doubt occasional attacks of it will be brought on by great errors as to diet, as is also the case with attacks of epileptic convulsions. But in both these maladies there is in most patients a constitutional tendency to them, which will bring them on at certain intervals whatever care is taken. A reasonable restriction as to food will prolong this interval, but the return of the attacks is a species of necessity and cannot be wholly prevented.

There are other conditions of the digestive organs, occurring either as insulated attacks or at the beginning or in the course of other diseases, which require notice, and they often present questions of treatment difficult to determine. Thus, severe and painful spasm may seize the stomach from the presence of undigested food, or from food of an improper quality or quantity. This spasmodic state may be regarded as the result of an abortive or imperfect attempt to vomit—just as cramp will sometimes seize the calf of the leg, or some other muscle, as a consequence of an attempted voluntary motion, instead of the normal voluntary contraction. In this state of things there are two modes of treatment, either of which may be successful or either of them fail, so that it is not always easy to decide between them; either by an emetic or ipecac, which shall substitute the normal effort to vomit for the spasmodic condition which seems, as it were, to grasp the offending contents in a close embrace; or by an opiate which quells the spasm, and thus brings the stomach into a condition that enables it to empty itself in the natural way, or suffer an emetic or cathartic to do this without opposition. When the spasm and pain is moderate, intermittent with long intervals, and accompanied by nausea and efforts to vomit, especially if we know that the food has been recently taken, in large quantity and very indigestible—an emetic guarded by an opiate and some aromatic, may be employed safely.* But if the spasm be fixed and violent, and the food has been taken some time before, so as to be partially digested and be passing into the intestines before the attack begins, a full opiate, repeated immediately if rejected, and at intervals, till the spasm is relieved, seems to be the proper method.

An analagous state of things occurs in the large intestine, giving rise to the different forms and degrees of colic. Colic probably originates in an effort to carry forward and discharge something from the bowels—either fæces, food or flatus —which is ineffectual and passes into spasm. Here, too, relief may be obtained in two ways, either by a cathartic, which substitutes the normal peristalic action for the morbid condition, or by an opiate, which quiets that condition, leaving the offending matters, after a period of rest, to be evacuated naturally or by a cathartic. When the attack is moderate, the cathartic practice may be employed, but in a large proportion of cases the opiate is better, or at any rate easier to the patient. Attacks of this description are very frequently met with, varying in degree from those which are well marked and formidable, to those so mild as to be simply very uncomfortable. The treatment will, of course, vary with the intensity. A dose of castor oil, or of some of the aromatic and stimulating purgatives, such as tincture of aloes and myrrh, or compound tincture of senna, or an operative enema, aided by external applications, are often quite sufficient, whilst at other times large doses of laudanum or other opiates became indispensable. It is acting on the safe side to prefer the latter method to the former. It certainly sometimes happens that the attempt to remove the trouble by cathartics converts a moderate and simple case into a severe and complicated one. The stomach is nauseated by the medicines administered and rejects them, or the whole canal is thrown, by the efforts excited, into a state of high irritation, and a train of distressing

* R. Inf. menth. pip. vel cinnam., two ounces; pulv. ipecac., two scruples;—zinc. sulph., one scruple; tinct. opii, thirty drops. Mix. One half to be taken at once and repeated.

and sometimes alarming symptoms follows. When dependence is placed upon opiates, it is better at first to give a little more than is absolutely necessary, than a little less. An under dose may require to be repeated, and thus in the end a larger quantity be taken than if a full dose be given at once.

There are some persons whose organs of digestion are extremely susceptible to all medicines, in whom the attempt to evacuate them at any period of disease, and sometimes even in health is productive of great irritation, indicated by nausea, vomiting, colicky pains, ineffectual retchings, and great restlessness and prostration. By some practitioners, the evacuation of the bowels is regurded of such indispensable necessity, that the attempt is repeated and persisted in, as I think, sometimes to the manifest injury, certainly to the great annoyance of the patient. It is seldom so essential a point as to render this advisable. Such a condition of the canal necessarily lessens the ability to contend with whatever disease may be present, and is a greater evil than the presence in it of the substances it is attempted to remove.

In such constitutions, when we are acquainted with them, it is better to trust the case wholly to the mildest palliatives, and soothing external applications, emptying the bowels only by enemeta. By this plan the disease may run through its natural course without evoking that tendency to gastric and intestinal irritation which is peculiar to the patient. Where the constitution is unknown, and this state of things is brought on by the ordinary course of treatment, as soon as it manifests itself all active interference should be suspended, and the disturbance that has been created may then spontaneously subside. The subsidence will be sometimes aided by articles grateful to the taste and gently stimulating to the stomach, such as some of the essential oils dissolved in alcohol—the oils of worm-wood, of checkerberry, of cinnamon, of peppermint, etc. Small quantities of soda combined with these, and effervescing mixtures in small and frequently-repeated doses, as of soda and lemon juice, will often be of service, as will also small quantities of brandy slightly diluted. The effect of opiates is more uncertain. A grain of solid opium or more is occasionally successful, or a frequent repetition of very small quantities of morphia, black drop or the liquid extracts. All attempts at evacuating the canal, except by enemata, should be avoided; irritating external applications are at best useless, and those only of a soothing kind will be of any avail, such as warm poultices to the abdomen and fomentations of water, hops, chamomile, etc.

The conditions here referred to as objects of attention are not in themselves the disease, but are accidents which may occur in any disease. Their occurrence, however, is an event of importance in the progress of the case in which it takes place, and may seriously interfere with its favorable course.—The violence of this secondary affection is, however, by no means any measure of the severity of the primary, but rather of a peculiarity in the patient himself. It may occur in even so light a disease as a severe cold, and, in the same person, is less likely to occur in a very grave attack than in one of a moderate character. The same thing is not unfrequently observed as to other constitutional peculiarities. They continue to exhibit themselves in cases of slight disease, but a very severe one, seems, as it were, to reduce all constitutions to nearly the same level, and to over-ride tendencies which manifest themselves on ordinary occasions. Thus, it is a matter of common observation that nervous and hysterical patients, who are abundantly troubled with their peculiar symptoms during trifling ailments, and even in their ordinary health, become tranquil, quiet and entirely free from them, in aggravated and especially in mortal diseases.

Somewhat related by certain affinities to the cases which have been considered, in another condition that may occur in almost any period of disease, in connection with the attempt to evacuate the alimeniary canal; I mean what is popularly called a "stoppage of the bowels." This is a prominent characteristic of an attack of ileus, and is then at once accompanied by other characteristic symptoms, but I refer more particularly here to its occurrence where there has been no preceding indication of any local difficulty in the bowels themselves. In a case where these organs have been acting very well, there is a sudden failure in the operation of a purgative; a more active one is administered, which is still resisted, and we find that the canal is closed. Perseverance in the attempt to open it only agravates the difficulty and brings on a state of general irritation, such as has been above described. Very powerful cathartics will sometimes force their way through and give relief, but not generally; and the case may assume all the formidable characteristics of ileus and prove fatal. The local state of the parts concerned cannot always be determined. In those which terminate in death, there is usually found some of those mechanical impediments which are enumerated as the causes of ileus, such as introsusception, diverticula, internal hernia, stricture, etc. But in those where the bowels finally give way, we can only conjecture the state of things. It may be simple torpidity, inflammation, or spasm of some part of the tract of the intestines.

The essential object of treatment was formerly judged to be the procuring of discharges. Because relief always followed the opening of the bowels, it was inferred that this was the *sine qua non*, and the thing to be accomplished at all events. But as it was found that in fatal cases a state of disease was revealed which no purgatives could have removed, it was quite as reasonable to judge that the "stoppage" in those recovering was dependent upon an entirely different condition of parts; that this condition gave way and then discharges took place, and that seemed to be the cause, which was in fact only the consequence. A practice in conformity with this view is now generally regarded as most judicious. Wherever there is a decided closure of the intestines, we are to cease active interference so far as this particular object is concerned. Where there is no pain or other indication of actual disease, we are simply to stand aloof, and by and by, in a vast proportion of cases, the difficulty is spontaneously resolved. Where there is pain, tormina, swelling the other symptoms of mechanical obstruction, and ineffectual efforts on the part of the intestine, all active symptoms are to be quelled by opium, and the parts are to be kept under its full influence till the patient dies or the bowels act of their own accord or by enemata. This course not only is attended by far less suffering in cases necessarily mortal, but by the more frequent recovery of cases which, under the aggressive form of treatment, seem likely to have come to the same termination.

I would remark, with regard to all these different states of the canal which have been spoken of, that in looking back upon past experience, I am persuaded that their occurrence is far less frequent, the recovery from them far more speedy, and the suffering of the subjects very much less, under the palliative treatment, than the persevering and active medication which it was formerly judged necessary to adopt.—*Bost. Medical Journal.*

